# Supplementary material for: An exploratory study on the handwritten allographic features of multi-ethnic population with different educational backgrounds
Source: PLoS One. 2022 Oct 7;17(10):e0268756. doi: 10.1371/journal.pone.0268756 (PMC9544031; doi:10.1371/journal.pone.0268756)
Supplement: Attachment — Submitted filename: Responses to Reviewers.docx [file pone.0268756.s003.docx]

Dear Editors and Reviewers of PLOS ONE,

Thank you for your kind review and thoughtful comments. We appreciate the constructive feedback and have carefully taken the comments into consideration in preparing this manuscript. The following summarised our response to the comments:

Reviewer #3

| Comments | Responses |
| --- | --- |
| The authors have improved the manuscript. They inserted the data on examination of handwritten samples and on participants. | Thank you. |
| However there are still some aspects that need revision. Please include the inter-rater reliability between forensic coders. This should be presented in the manuscript. The limitations are still not elaborated. | Procedure of the examination and coding of the handwriting features was described in the manuscript in which the handwriting samples were examined in two different occasions by two teams of researcher independently, and their results were validated by a certified forensic document examiner. In this study, only the data validated by the document examiner was considered in interpretation and data analysis (Line 161-162).  As for the inter-rater reliability among the coders as suggested by the reviewer, a greater number of coders shall be proposed to involve multiple raters examining and coding the same set of handwriting samples, and this was not conducted in the scope of the current study. The inter-rater reliability study would be more useful upon the determination of specific allographic features for discrimination among the groups as demonstrated in the current study, perhaps to involve also expert and non-experts as a future recommendation. Such limitation and future recommendation were included in the revised manuscript (Line 407-413). |
| Many factors can differentiate the forms of letter examined by the researchers, not only education and nationality. These factors such as intelligence or perceptive/manual impairments were not controlled in the present study. The researchers should be aware of these potential factors, particularly in the situation while the sample size is small. | The inclusion and exclusion criteria of the subjects of this study was included in the revised manuscript (Line 92-94). In general, they were university students who are mentally challenged, no physical disabilities and well-versed in reading and writing in English Language.  The limitation on the sample size was also included in the revised manuscript (Line 240-241, 418-420), deserving further investigation in the future with greater number of participants. |
| The list of references is very short. Please explain the impact of these potential factors on handwriting in Discussion and say why these factors were not controlled as well as enrich the literature on this topic. | Additional references with related information were included in the revised manuscript (Line 397-401).  The potential factors which could affect the development of handwriting were also discussed in the revised manuscript (Line 401-406). |

This manuscript is checked thoroughly. We really appreciate the efforts taken by the editor and reviewers to help us improve the manuscript, and we hope that we have addressed all your concerns in this manuscript.

Thank you.

Ahmad Fahmi Lim Abdullah, PhD

School of Health Sciences, Universiti Sains Malaysia, 16150 Kubang Kerian, Kelantan, Malaysia. Email: [fahmilim@usm.my](mailto:fahmilim@usm.my); Tel: +609-7677596; Fax: +609-7677515.
