# Supplementary material for: An exploratory study on the handwritten allographic features of multi-ethnic population with different educational backgrounds
Source: PLoS One. 2022 Oct 7;17(10):e0268756. doi: 10.1371/journal.pone.0268756 (PMC9544031; doi:10.1371/journal.pone.0268756)
Supplement: Attachment — Submitted filename: Responses to Reviewers.docx [file pone.0268756.s004.docx]

Dear Editors and Reviewers of PLOS ONE,

Thank you for your kind review and thoughtful comments. We appreciate the constructive feedback and have carefully taken the comments into consideration in preparing this manuscript. The following summarised our response to the comments:

Editor

| Comments | Responses |
| --- | --- |
| 1) Several reviewers previously raised methodology concerns, including sample size, statistical analysis, etc. Although this has been addressed in the study as being relevant for an exploratory study, please provide some additional justification for these issues. | Thank you for the comment. The justification on the sample size determination and choice of statistical analysis was included in the revised manuscript.  Line 88-91 - “Sample size was calculated through two proportion formula by considering the letter “U” with introductory stroke [4], 25% of the Polish (0% for English) possessed the handwritten feature. With 80% of power study, 5% of risk error, and 10% of non-response rate, 30 writers were collected from each group.”  Line 386-388 - “By further including the multinomial logistic regression model in this study, it allowed for the understanding of which primary education system has a higher probability of shaping specific handwritten allographic features, adjusted with confounding variables.” |
| 2) There are some outstanding language issues which, if rectified, would strengthen this submission. For example, some information is missing or unclear, e.g., lines 245-247, line 399, as well as others. Therefore, we highly encourage a thorough proof-reading prior to resubmission. | Thank you for the comments.  Line 245-247 was revised to include the percentage of the power of study and its description as follows:  “Handwritten feature with low powder of study (<70%) is suggested for further investigation with greater sample size, in this case the uppercase "A" with three individual strokes. For the current study, only those statistically significant features with adequate power (in this case greater than 78%) are discussed.” (Line 241-244).  Line 399 was also revised to “Previous studies had suggested foreign influence toward the formation of handwriting, particularly in countries that have welcomed a huge number of immigrants in the past.” (Line 391-392). |
| 3) Please also reconsider a rephrasing of your interchangeable use of 'race' and 'nationality' (some guidelines for this may be found here - https://jamanetwork.com/journals/jama/fullarticle/2783090). | Thank you for the comment. In this study, the subjects were from the three main ethnicity groups, and they are from the same country (same nationality). In the revised manuscript, the term “ethnicity” was used in the results and discussion. |

Reviewer #4

| Comments | Responses |
| --- | --- |
| The conclusion can include more application areas for the study. The paper is well structured. Research methodology is adequately developed. | Thank you for the comment. The conclusion was added with “Nonetheless, the examination of questioned handwriting can still be expanded to retrieve useful information thus narrowing down the possible groups of potential writers, in this case, providing information on the educational backgrounds.” to support the application for the study. |

This manuscript is checked thoroughly. We really appreciate the efforts taken by the editor and reviewers to help us improve the manuscript, and we hope that we have addressed all your concerns in this manuscript.

Thank you.

Ahmad Fahmi Lim Abdullah, PhD

School of Health Sciences, Universiti Sains Malaysia, 16150 Kubang Kerian, Kelantan, Malaysia. Email: [fahmilim@usm.my](mailto:fahmilim@usm.my); Tel: +609-7677596; Fax: +609-7677515.
